# Photolithographic patterning of viologens containing styrene groups†

**DOI:** 10.1039/d3ra02287k

**Published:** 2023-05-31

**Authors:** Radosław Banasz, Monika Wałęsa-Chorab

**Affiliations:** a Faculty of Chemistry, Adam Mickiewicz University in Poznań Uniwersytetu Poznańskiego 8 61-614 Poznań Poland mchorab@amu.edu.pl

## Abstract

A simple method for the patterning of styrene derivatives for electrochromic applications is presented. Novel viologen derivatives containing styrene groups were used in the formation of patternable electrochromic films. The patterning was done *via* photopolymerization and it shows the possibility of the use of styrene derivatives for the preparation of electrochromic patterns.

## Abstract 2

A method for the formation of patterns for large area electrochromic segmented displays *via* the use of styrene derivatives was developed.

## Graphical abstract

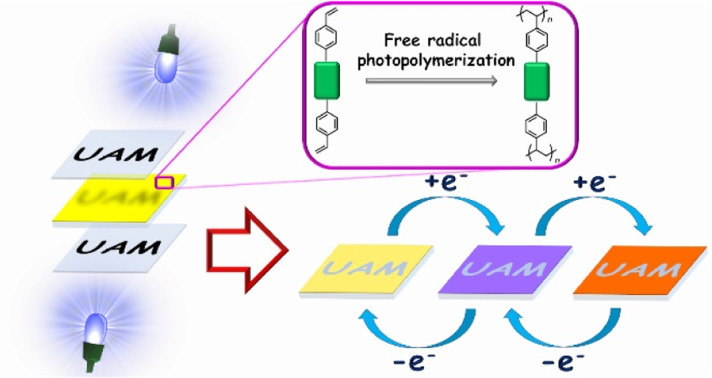

Electrochromic segmented displays are attracting attention owing to their potential application in the design of intelligent displays.^1^ Such devices present a relatively simple display mode in which numbers and letters of fixed graphics can be displayed.^2–4^ The assembly of segmented displays usually requires the formation of an electrochromic pattern on the working electrode. This can be done by electrode patterning strategy or active material patterning.^5^ In the case of electrochromic segmented displays, the required size of pixel depends on the application conditions. When the display is used as an electronic screen, the required resolution is in the order of micrometers, whereas in the case of large area displays such as electronic billboards, it can be in the order of millimeters or centimeters.

The patterning of functional materials is one of the most important steps in the fabrication of functional devices.^6,7^ Different patterning methods have been developed,^8^ and they can be divided into the following groups: photolithography,^9^ printing methods,^10,11^ template-assisted methods,^12^ and other patterning methods, such as the use of self-assembled monolayers,^13^ hydrophilic/hydrophobic differences,^14^ and laser etching.^15^ To obtain photopatterned thin films, different precursors have been used. For example, Kim *et al.* used methacrylate-functionalized poly(3,4-alkylenedioxythiophene)s to obtain large-area electrochromic films. Other examples include xanthane precursors^16^ and poly(*p*-xylenetetrahydrothiophenium) chlorides,^17^ which upon irradiation undergo a partial photoelimination reaction that leads to the formation of insoluble poly (*p*-phenylenevinylene)s. The formation of photopatterns is also possible using the cationic ring opening polymerization (CROP) of oxetanes^18,19^ and oxiranes^20^ or through ring-opening metathesis polymerization (ROMP).^21^ The novel approach of optical lithography is based on the formation of photocurable materials using polystyrene.^22^ Styrene derivatives are commonly used in thermal crosslinking^23–26^ where the formation of a pattern is facilitated by the use of printing or template-assisted methods, but to the best of our knowledge, the formation of functional patterns using styrene derivatives *via* photopolymerization has never been reported before.

Two linear viologens containing benzothiadiazole (1) and benzoselenadiazole (2) cores were designed and obtained *via* a two-step synthesis (Fig. 1). The benzothiadiazole and benzoselenadiazole building blocks have been chosen as bridging groups owing to their interesting electrochemical and electrochromic properties.^27–29^ In the first step, the 4,7-dibromo derivatives of benzothiadiazole and benzoselenadiazole were coupled with 4-pyridinylboronic acid to give the dipyridyl derivatives (A and B). The experimental procedure for A has been described in literature.^30^ Subsequently, the microwave-assisted alkylation reaction between the intermediates (A and B) and 4-vinylbenzyl chloride resulted in the formation of their respective chloride salts, which were subsequently converted to hexafluorophosphate salts *via* anion exchange. This was done to increase the solubility of the viologens in common organic solvents,^31^ since the chloride salts were hard to dissolve in any organic solvent.

**Fig. 1:**
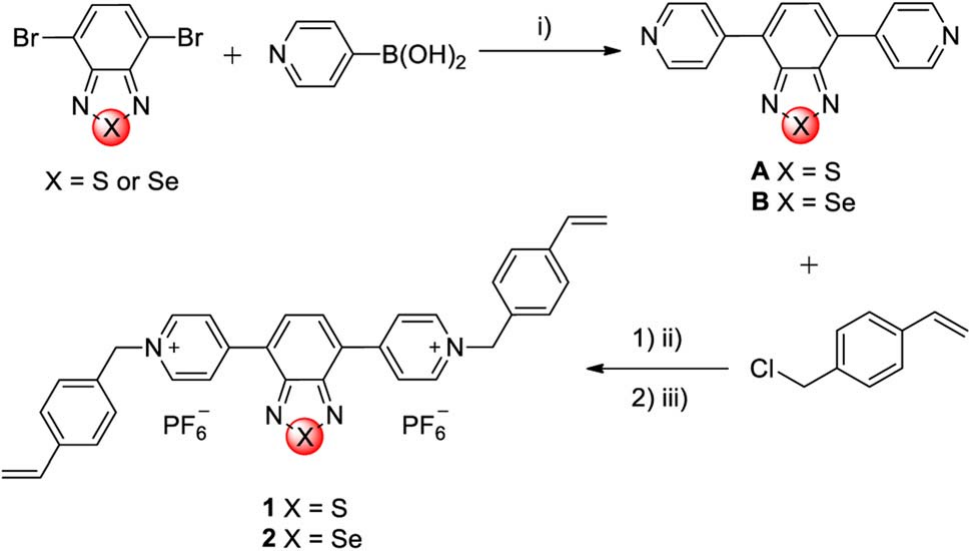
Synthetic scheme of investigated viologens. Reaction conditions: (i) Pd(PPh_3_)_4_, K_2_CO_3_, 1,4-dioxane/H_2_O, 90 °C, 96 h; (1) (ii) acetonitrile, 135 °C, 2 h, microwave reactor; (2) (iii) NH_4_PF_6(aq)_, 20 °C, 24 h.

The prepared compounds were characterized by NMR spectroscopy and HR-ESI-MS method. The benzothiadiazole and benzoselenadiazole building blocks were chosen as bridging groups owing to their interesting electrochemical and electrochromic properties.

The electrochemical properties of viologens 1 and 2 and their respective polymers poly-1 and poly-2 were examined by cyclic voltammetry. This was done in an anhydrous and deaerated 0.1 M solution of tetrabutylammonium hexafluorophosphate (TBAPF_6_) in acetonitrile as a supporting electrolyte using a platinum working electrode in the case of viologens in solution or polymer-coated ITO electrode, platinum wire as a counter electrode and silver wire as a pseudo-reference electrode. Ferrocene was used as an internal (in the case of viologens) or external (for the polymers) standard. The voltammograms were calibrated against a ferrocene/ferrocenium couple (*E*_1/2Fc/Fc+_ = 0.40 V *vs.* SCE in 0.1 M TBAPF_6_ in acetonitrile).^32^ The cyclic voltammograms are shown in Fig. 2 and electrochemical data are listed in Table 1.

**Fig. 2:**
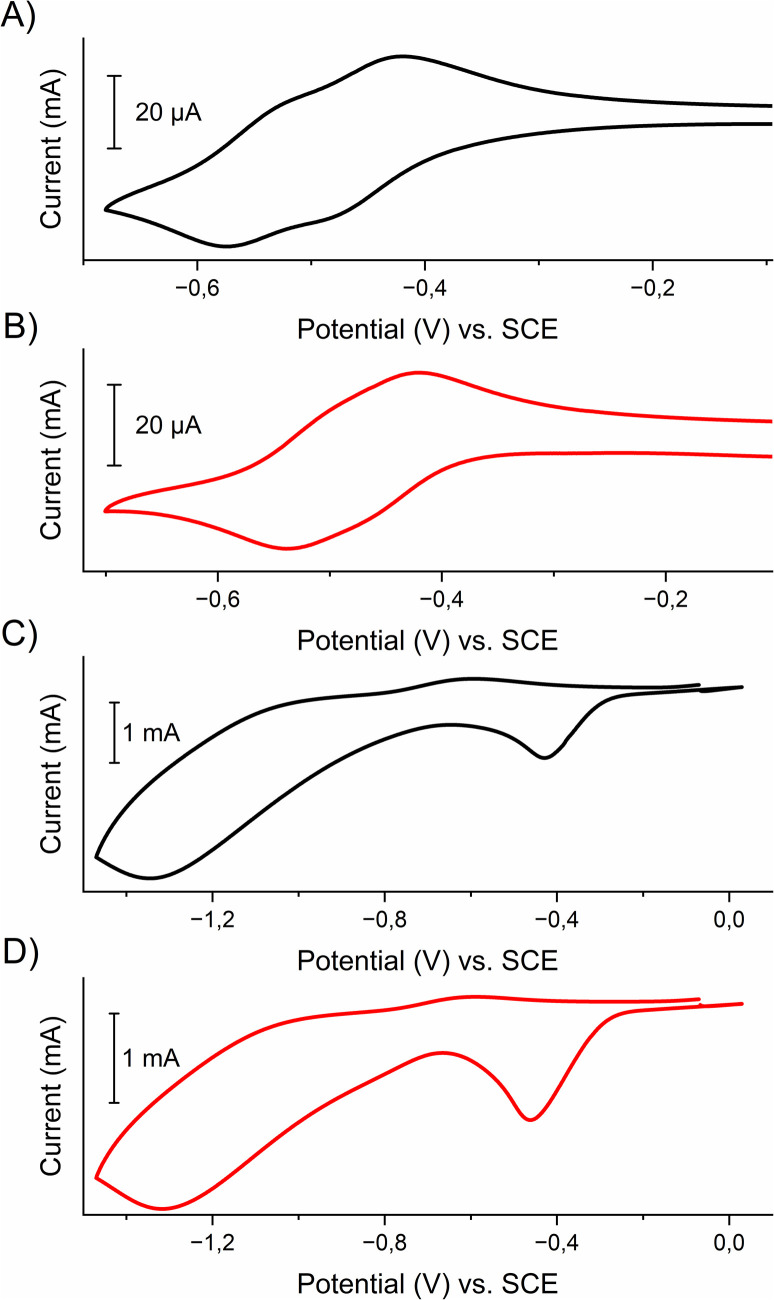
Cyclic voltammograms of viologens (A) 1 (black) and (B) 2 (red) and polymers (C) poly-1 (black) and (D) poly-2 (red) immobilized on ITO electrode measured in an anhydrous and deaerated 0.1 M solution of TBAPF_6_ in acetonitrile as a supporting electrolyte at 100 mV s^−1^.

**Table 1:** Electrochemical data of monomers 1 and 2 and polymers poly-1 and poly-2

Compound	*E*_pa_ (V)	*E*_pc_ (V)	*E*_1/2_ (V)	Δ*E* (mV)
1	−0.42	−0.48	−0.45	60
	−0.53	−0.57	−0.55	40
2	−0.42	−0.46	−0.44	40
	−0.50	−0.54	−0.52	40
Poly-1	−0.59	−0.43	−0.51	160
	−1.00	−1.35	−1.18	350
Poly-2	−0.59	−0.46	−0.53	130
	−0.97	−1.32	−1.15	350

Both viologens undergo two step reductions with similar *E*_1/2_ values. For example, in the case of viologen 1 the first reduction process occurs at *E*_pc1_ = −0.48 V, which is related to the electroreduction of the dication form of the viologen to radical cation, while the second reduction process at *E*_pc2_ = −0.57 V corresponds to the formation of the neutral form.

Similar electrochemical processes were observed for the polymers poly-1 and poly-2. Both polyviologens exhibited two cathodic peak potentials, which were located at −0.43 V and −1.35 V for poly-1 and −0.46 V and −1.32 V for poly-2. The reduction processes were found to be irreversible, which was probably caused by the kinetic charge trapping in the polymer.^33^

The spectroelectrochemical properties of the polymers were further investigated in order to investigate their usefulness as active materials in electrochromic devices. Fig. 3 shows that poly-1 exhibited a pale yellow color in the dication state and an absorption band with a maximum at 360 nm. In the case of poly-1, when a negative potential in the range from 0 V to −0.3 V was applied, a new absorption band was formed with a maximum at 430 nm, which corresponds to the formation of the radical cation form.

**Fig. 3:**
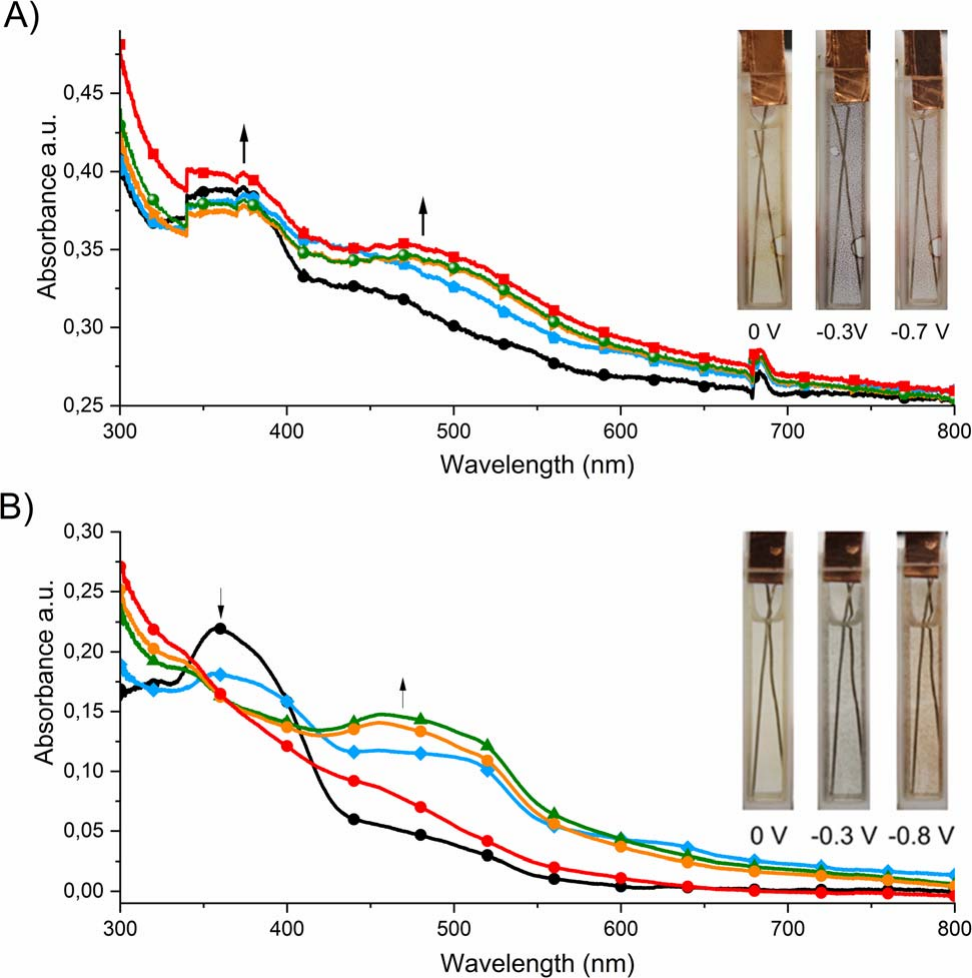
Spectral changes of (A) poly-1 and (B) poly-2 measured in anhydrous and deaerated 0.1 M solution of TBAPF_6_ in acetonitrile as the supporting electrolyte with applied potentials of 0 V (black), −0.3 V (blue), −0.7 V (olive), −0.8 V (orange) and −1.2 V (red) *vs.* Ag/Ag^+^ held for 30 s per potential. (Inset) Photographs of polyviologen in dication form (left), radical cation (middle) after applying the potential of −0.3 V and neutral form (right) after applying the potential of −0.7 V for poly-1 (A) and −0.8 V for poly-2 (B).

Further decrease in the applied potential (from −0.3 V to −0.7 V) caused a shift of the absorption band to longer wavelengths (470 nm) and an increase in its intensity. These bands are responsible for the color changes from yellow to blue to red. Similar spectroelectrochemical changes were observed for poly-2. The absorption band derived from the dication form was located at 360 nm, which disappeared when a negative voltage (up to −0.3 V) was applied. Simultaneously a new band with a maximum at 475 nm was formed, which can be assigned to the formation of the radical cation of poly-2. A further reduction of polymer caused a shift of the absorption band to lower wavelengths (up to 455 nm). The visible color changes of the polymer were from yellow to blue to red.

The thin films of polymers (poly-1 and poly-2) were easily obtained by the photopolymerization of the appropriate monomer (1 or 2) (Fig. 4). A solution of the appropriate viologen and 2,2-dimethoxy-2-phenylacetophenone (DMPA) as a photoinitiator in acetonitrile were spray-coated on the surface of a glass slide. The spray-coating method was used as it is known to be the simplest method that allows to coat substrates of different shapes. The substrates with thin layers obtained this way were placed between two glass slides with the marked inscription. The samples were exposed to UV light (365 nm) using a PhotoCube multi-wavelength photoreactor for 5 minutes. Due to the fact that the exposure of the formed monomer layer to UV light occurs from both sides (from the top and bottom) in the photoreactor two identical patterns were used to cover the sample from the top and the bottom. To obtain homogenous irradiation four LED panels were used and the light intensity was set to be 100%. The substrates were washed with acetone and acetonitrile to remove unreacted monomers and low molecular weight oligomers, and the pattern formed immediately on the glass slides according to the photomask pattern (Fig. 5A and S1†).

**Fig. 4:**
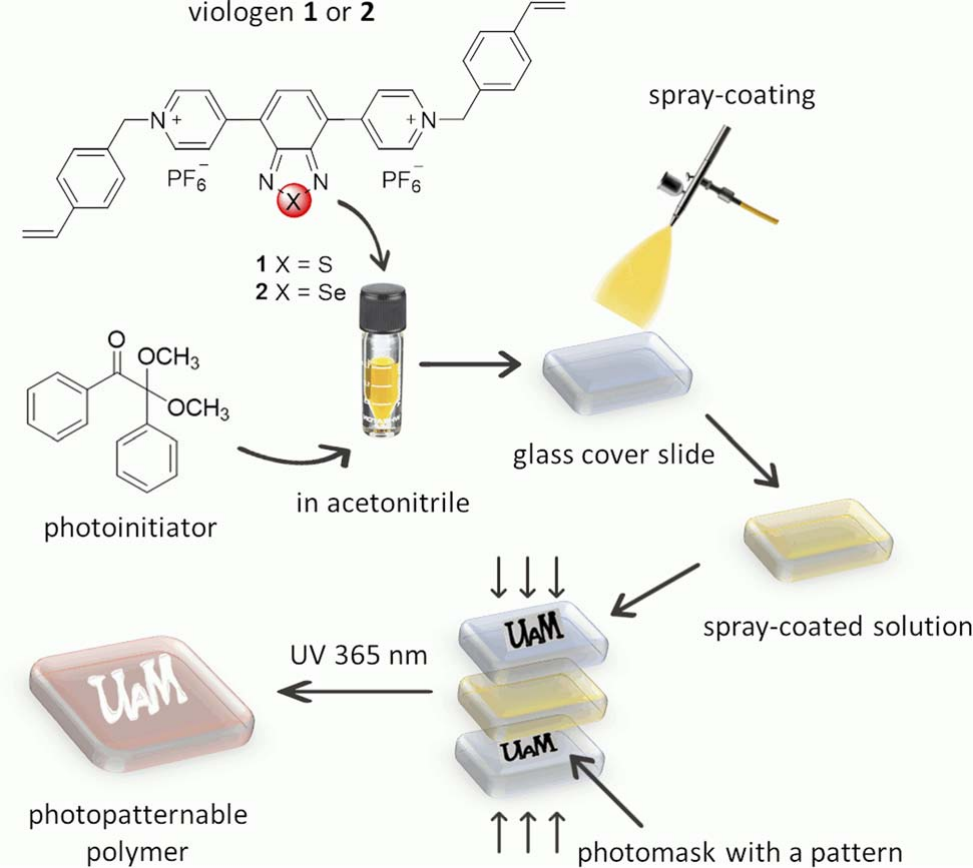
Schematic of the fabrication process of patterned polyviologen layers.

**Fig. 5:**
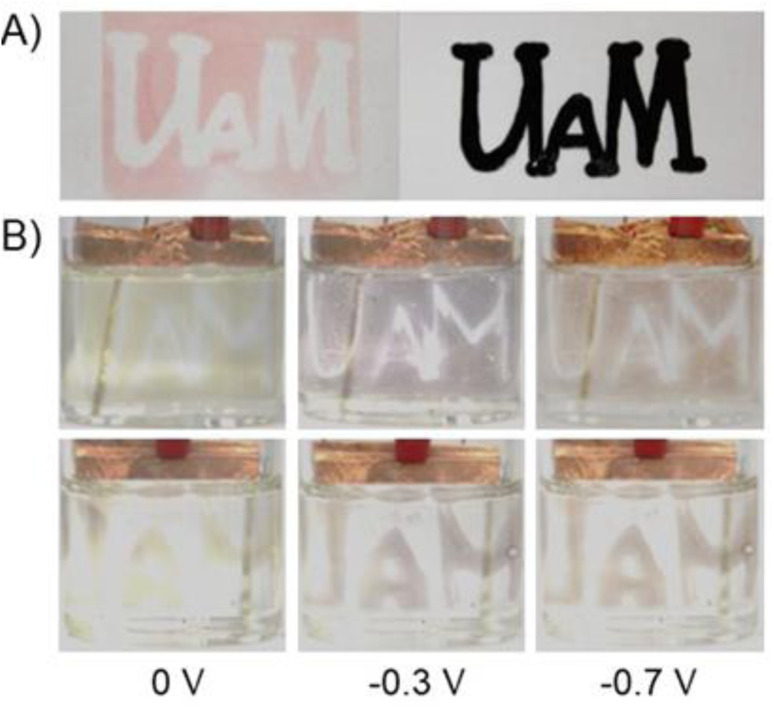
(A) Photograph showing the photopatterns of a logo of UAM photolithographically immobilized on a glass substrate (left) and photomask used for the photopatterning (right). (B) Electrochromic behaviour of patternable poly-1 coated onto ITO glass in different redox states: dication form (left), radical cation after applying the potential of −0.3 V (middle) and natural form after of −0.7 V.

Upon irradiation DMPA undergoes photolysis to form radicals^34^ that are able to initiate the polymerization reaction of styrene groups. As can be seen, polymerization occurred only in places that were irradiated, while the areas covered by the photomask remained uncoated. The formation of polymer was observed although the irradiation proceeded under normal atmosphere in the presence of oxygen. The polymerization probably occurs according to a typical free-radical polymerization mechanism.^35,36^ After formation of the pattern, the color of the film was red but it changed to yellow after immersion in the solution of supporting electrolyte. The red color of the polymer was probably caused by the partial degradation of PF_6_^−^ upon UV irradiation^37,38^ and formation of F^−^ anions that remained in the polymer layer and balanced the charge of viologens. When the film was immersed in the electrolyte solution, the exchange of F^−^ ions with PF_6_^−^ probably occurred leading to the color change of the polymer. The electrochromic properties of both patternable polymers were investigated (Fig. 5B and S2†).

To investigate the influence of the wavelength of irradiation on the polymerization process of styrene derivatives, the polymerization was carried out by irradiating with light of different colors: UV (365 nm), violet (395 nm), blue (457 nm), cyan (500 nm), green (523 nm), amber (595 nm), and red (625 nm). The same amount of monomer was spray-coated on the substrates. This was provided by spray-coating all substrates at the same time, and the absorbance of monomers on each substrate was investigated by UV-Vis spectroscopy. This confirmed that the absorbance of the monomers deposited on all substrates was similar. The samples were exposed to light of different color for 5 minutes. To obtain homogenous irradiation, four LED panels were used and the light intensity was set to 100% each time.

After washing and immersing in the solution of PF_6_^−^ anions for 10 minutes the absorbance of the polymers was measured. It was found that with the shift of the wavelength of irradiating light to longer values the absorption of the polymer at the substrate decreased (Fig. 6). The high absorption value exhibited by the polymers when irradiated with UV light indicated that the polymerization was most effective when irradiated with the UV light. Irradiation with violet, blue, and cyan light resulted in the partial polymerization of monomers, whereas when substrates were irradiated with green, amber, or red light no polymerization was observed.

**Fig. 6:**
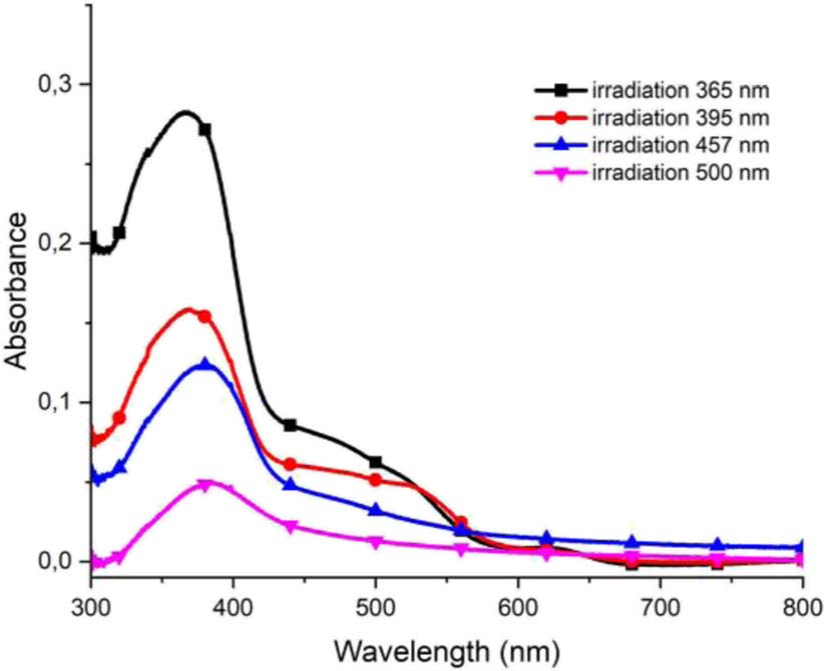
UV-Vis spectra of polymer poly-1 obtained after irradiation with light of different wavelengths.

We further investigated the usefulness of this type of photopolymerization for the preparation of smaller patterns. For this, a pattern of different sizes was prepared and photopolymerization was done. As seen in Fig. 7, the patterning of a square of minimum 3 mm × 3 mm size was possible, although some shadowing was observed. The patterning of lower sizes was not possible and as a result this polymerization is more appropriate for preparing large-area electrochromic devices.

**Fig. 7:**
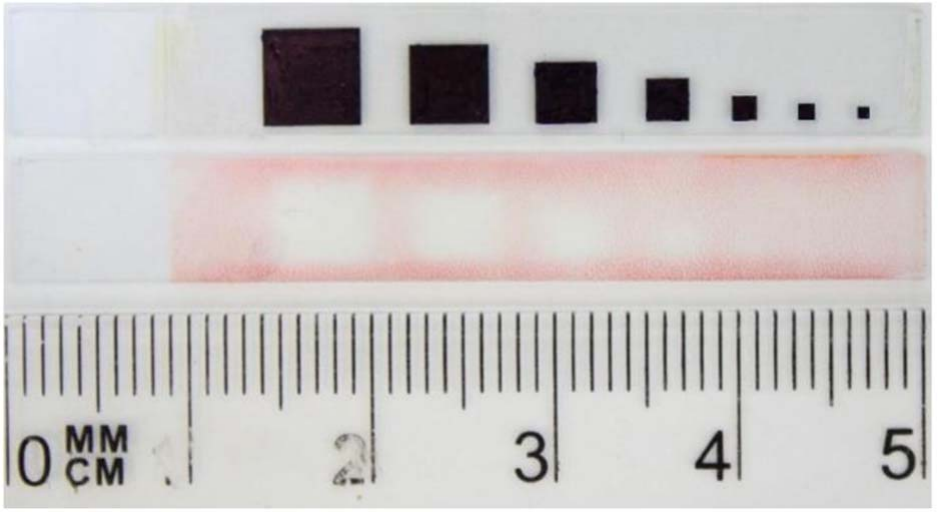
Photopattern containing patterns of different size photolithographically immobilized on a substrate and photomask used for the photopatterning.

## Conclusion

In conclusion, the usefulness of the styrene derivatives in the formation of patterns of active materials has been shown and it provided an alternative way for the formation of patterns for large-area electrochromic segmented displays. The presented photolithographic process is simple and an inert atmosphere is not required to polymerize the styrene groups. The process can be used for different styrene-appended molecules and future work will be focused on the improvement of the electrochromic properties of polymers obtained *via* photopolymerization.

## Author contributions

Radosław Banasz: investigation, writing, funding acquisition, project administration; Monika Wałęsa-Chorab: conceptualization, methodology, investigation, resources, writing – review & editing, visualization, supervision.

## Conflicts of interest

There are no conflicts to declare.

## Supplementary Material

RA-013-D3RA02287K-s001
