# Supplementary material for: Photolithographic patterning of viologens containing styrene groups
Source: RSC Adv. 2023 May 31;13(24):16206–10. doi: 10.1039/d3ra02287k (PMC10230511; doi:10.1039/d3ra02287k)
Supplement: RA-013-D3RA02287K-s001 [file RA-013-D3RA02287K-s001.pdf]

Electronic Supplementary Material (ESI) for RSC Advances.
This journal is © The Royal Society of Chemistry 2023

Photolithographic patterning of viologens containing styrene groups

Radosław Banasz, Monika Wałęsa-Chorab*

Faculty of Chemistry, Adam Mickiewicz University in Poznań, Uniwersytetu Poznańskiego 8, 61-614 Poznań, Poland, e-mail: mchorab@amu.edu.pl

Table of content

Experimental procedures.. 3

Figure S1. Photograph showing the photomasks used for the photopatterning of a logo of UAM on the ITO glasses.. 5

.... 6

Figure S2. Electrochromic behaviour of patternable **poly-2** coated onto ITO glass in different redox states: dication form (left), radical cation (middle) after applying the potential of -0.3 V and natural form after of -0.8 V.. 6

Figure S4. ^{13}C NMR spectra of compound **A** in $CDCl_3$.. 7

Figure S5. ^{1}H NMR spectra of compound **B** in $CDCl_3$.. 7

Figure S6. ^{13}C NMR spectra of compound **B** in $CDCl_3$.. 8

Figure S7. ^{1}H NMR spectra of compound **1** in CD_3CN.. 8

Figure S8. ^{13}C NMR spectra of compound **1** in CD_3CN.. 9

Figure S9. ^{1}H NMR spectra of compound **2** in CD_3OD.. 9

Figure S10. ^{13}C NMR spectra of compound **2** in CD_3OD.. 10

Figure S11. HR-ESI-MS spectra of compound **A**.. 10

Figure S12. HR-ESI-MS spectra of compound **B**.. 11

Experimental procedures

Spectroscopy

Spectroscopic measurements consisted in performing NMR spectra using the Bruker Avance 600 MHz apparatus. The tests were performed in deuterated solvents from Deutero GmbH, and the spectra were calibrated against the solvent signals ($CDCl_3$ = 7.26 ppm, CD_3CN = 1.94 ppm and CD_3OD = 4.78 ppm). HR-ESI-MS spectra were measured on a QTOF mass spectrometer (Impact HD, Bruker) in the positive ion mode.

Electrochemistry

Electrochemical measurements were done using a Bio-Logic VSP potentiometer. Monomers were dissolved in anhydrous acetonitrile electrolyte solution 0.1 M $TBAPF_6$. A platinum electrode was used as a working electrode, a platinum wire as the counter electrode, and a silver wire electrode was used as the pseudoreference electrode. The polymers were measured using the ITO electrode as the working electrode, the pseudoreference electrode was silver wire and the platinum wire as counter electrode.

Spectroelectrochemistry

Spectroelectrochemical measurements were recorded using a potentiostat and a UV-vis-NIR spectrometer (Jasco V770) by stepwise increasing the applied potential and simultaneously measured the absorption spectra. The solution of monomers with a supporting electrolyte were tested using gold honeycomb electrode in quartz cuvette with the silver wire as a reference electrode. The thin film of the polymers on ITO electrode were measured in anhydrous acetonitrile electrolyte solution 0.1 M $TBAPF_6$ where silver wire was used as a reference electrode and platinum wire as the counter electrode.

Preparation of polymer thin films

ITO glasses (5 × 0.7 cm) or glass cover slides (5 × 2.5 cm) were previously sonicated in water and 2-propanol for 15 min and then blowed it with an airbrush to dry.[1] Afterwards they were exposed to an UV-ozone atmosphere for 20 min. The compound **1** or **2** (4 mg) and 2,2-dimethoxy-2-phenylacetophenone (~10% mol) were dissolved in a small amount of anhydrous acetonitrile. The solution was spray-coated on the substrate and the substrates were irradiated with a UV lamp at 365 nm for 5 min. To obtain homogenous irradiation four LED panels were used and the light intensity was set to be 100% at every time. Then the resulting films on glasses were rinsed with acetonitrile and acetone and they were air-dried.

Preparation of polymer pattern

The glass slide with the spray-coated thin layer of monomer **1** or **2** was placed between two glass plates with the marked inscription. Self-adhesive tape was used to hold the three glass cover slides overlapping each other and this setup was placed in the photoreactor and then irradiated at 365 nm for 5 min. To obtain homogenous irradiation four LED panels were used and the light intensity was set to be 100% at every time. The formed polymer pattern was left to dry after washing the glasses with acetonitrile and acetone to remove the monomer residue and other impurities.[2,3] For preparing of smaller patterns, the same regularly repeating design with different sizes was prepared and photopolymerization was done. The patterning of

square at the different sizes was used as a photomask to obtain photopatterns photolithographically immobilized on a substrate.

Investigation of polymer after irradiation with light of different wavelengths

The same amount of monomer **1** was spray-coated at the same time on the seven glass slides. Afterwards the each of spray-coated sample was irradiated with light of different colors according to the previously described procedure: UV (365 nm), violet (395 nm), blue (457 nm), cyan (500 nm), green (523 nm), amber (595 nm) and red (625 nm). The substrates containing the polymer were washed with dichloromethane and immersed in the solution of $TBAPF_6$ to ensure the anion exchange. After washing the substrates, the absorbance of obtained polymers were measured using UV-Vis.

All the electrochemical and spectroelectrochemical measurements as well as polymerizations were done several times to ensure reproducibility of the results.

Synthesis

4,7-di(4-pyridyl)-2,1,3-benzothiadiazole (A).[4]

4-pyridylboronic acid (0.96 g, 7.81 mmol), 4,7-dibromobenzothiadiazole (1.00 g, 3.40 mmol) and potassium carbonate (1.88 g, 13.60 mmol) were solubilized in 1,4-dioxane/water mixture (40 mL, 3/1, v/v) under an argon atmosphere. The solution was degassed with argon for 15 min and then tetrakis(triphenylphosphine)palladium(0) (0.39 g, 0.34 mmol) was added. The mixture was stirred and heated under reflux at 90°C in an inert atmosphere for 96 h. After cooling, the reaction mixture was extracted with dichloromethane and washed with water and brine. The organic phase was collected, dried over magnesium sulfate and the solvent was evaporated under vacuum. The crude product was purified by column chromatography on silica gel using dichloromethane/acetone (9:1) to give a product as a pale yellow solid (0.60 g, 61%). ^{1}H NMR (600 MHz, $CDCl_3$) δ 8.81 (d, *J* = 6.2 Hz, 4H), 7.94 (d, *J* = 1.0 Hz, 4H), 7.93 (s, 2H) ppm. ^{13}C NMR (100 MHz, $CDCl_3$) δ 150.40, 150.21, 144.08, 131.88, 128.42, 123.50 ppm. HR-ESI-MS *m/z* = [**A** + H]$^+$ calculated: 291.0699 found: 291.0703 (100 %).

4,7-di(4-pyridyl)-2,1,3-benzoselenadiazole (B).

Similar procedure to **A** was used: 4-pyridylboronic acid (0.97 g, 7.89 mmol), 4,7-dibromobenzoselenadiazole (1.00 g, 2.93 mmol) and potassium carbonate (1.62 g, 11.72 mmol) were solubilized in 1,4-dioxane/water mixture (70 mL, 3/1, v/v) under an argon atmosphere. The solution was degassed with argon for 15 min and then tetrakis(triphenylphosphine)palladium(0) (0.41 g, 0.36 mmol) was added. The mixture was stirred and heated under reflux at 90°C in an inert atmosphere for 96 h. After cooling, the precipitate was solubilized in dichloromethane and washed with water and brine. The organic phase was collected, dried over magnesium sulfate and the solvent was evaporated under vacuum. The crude product was purified by column chromatography on aluminium oxide using dichloromethane/acetone (9:1) to give a product as a light green solid (0.27 g, 27%).^{1}H NMR (600 MHz, $CDCl_3$) δ 8.79 (d, *J* = 6.1 Hz, 4H), 7.85 (d, *J* = 6.1 Hz, 4H), 7.77 (s, 2H) ppm. ^{13}C NMR (100 MHz, $CDCl_3$) δ 158.94, 150.23, 145.07, 133.82, 129.03, 124.11 ppm. HR-ESI-MS *m/z* = [**B** + H]$^+$ calculated: 339.0143 found: 339.0149 (100 %).

4,4’-(benzo)[c][1,2,5]thiadiazole-4,7-diyl)bis(1-(4-vinylbenzyl)pyridine-1-ium)) hexafluorophosphate(V) (1).

4-vinylbenzyl chloride (0.44 mL, 3.12 mmol) was slowly injected to the solution of compound **A** (0.20 g, 0.69 mmol) in deaerated acetonitrile (15 mL). The mixture was stirred and heated at 135°C for 2 h in microwave reactor. After cooling, the yellow solid was centrifuged, dissolved in water and then was added dropwise to the solution of ammonium hexafluorophosphate (0.60 g, 3.68 mmol). The reaction mixture was stirring overnight at room temperature and the resulting solid was centrifuged. The final product was obtained as a yellow solid by recrystallization from water (92.50 mg, 33%).^{1}H NMR (600 MHz, CD_3CN) δ 8.91-8.86 (m, 4H), 8.75-8.71 (m, 4H), 8.35 (s, 2H), 7.61-7.56 (m, 4H), 7.52-7.47 (m, 4H), 6.80 (dd, *J* = 17.6, 11.0 Hz, 2H), 5.91 (d, *J* = 0.9, 1H), 5.88 (d, *J* = 0.8 Hz, 1H), 5.77 (s, 4H), 5.36 (dd, *J* = 11.0, 0.8 Hz, 2H) ppm. ^{13}C NMR (100 MHz, CD_3CN) δ 153.83, 153.44, 145.45, 140.18, 136.76, 133.21, 132.25, 130.80, 130.62, 128.93, 128.13, 116.41, 64.84 ppm. HR-ESI-MS *m/z* = [**1** - $(PF_6)^-]^+$ calculated: 669.1671 found: 669.1663 (0.3 %).

4,4’-(benzo)[c][1,2,5]selenadiazole-4,7-diyl)bis(1-(4-vinylbenzyl)pyridine-1-ium)) hexafluorophosphate(V) (2).

Similar procedure to **1** was used: 4-vinylbenzyl chloride (0.15 mL, 1.06 mmol) was slowly injected to the solution of compound **B** (0.089 g, 0.26 mmol) in deaerated acetonitrile (12 mL). The mixture was stirred and heated at 135°C for 2 h in microwave reactor. After cooling, the yellow solid was centrifuged, dissolved in water and then was added dropwise to the solution of ammonium hexafluorophosphate (0.43 g, 2.64 mmol). The reaction mixture was stirring overnight at room temperature and the resulting solid was centrifuged. The final product was obtained as a green solid by recrystallization from water (135.80 mg, 60%).^{1}H NMR (600 MHz, CD_3OD) δ 9.17-9.12 (m, 4H), 8.91-8.84 (m, 4H), 8.34 (s, 2H), 7.61-7.51 (m, 8H), 6.79 (dd, *J* = 17.6, 10.9 Hz, 2H), 5.94-5.84 (m, 6H), 5.34 (dd, *J* = 11.0, 0.8 Hz, 2H) ppm. ^{13}C NMR (100 MHz, $CDCl_3$) δ 158.82, 154.77, 145.56, 140.82, 137.12, 133.87, 132.67, 132.43, 130.52, 129.55, 128.42, 116.05, 65.08 ppm. HR-ESI-MS *m/z* = [**2** - $(PF_6)^-]^+$ calculated: 286.0734 found: 286.0725 (11%).

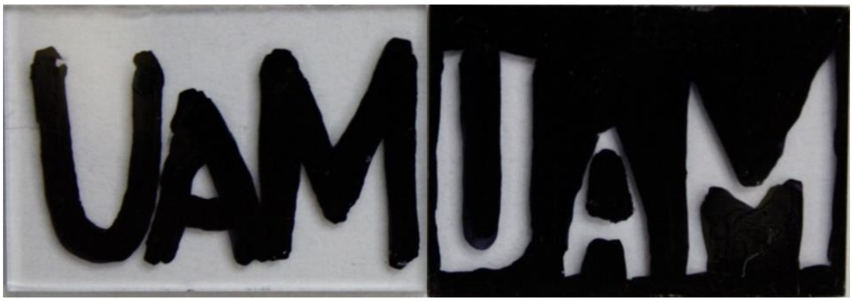

Figure S1. Photograph showing the photomasks used for the photopatterning of a logo of UAM on the ITO glasses.

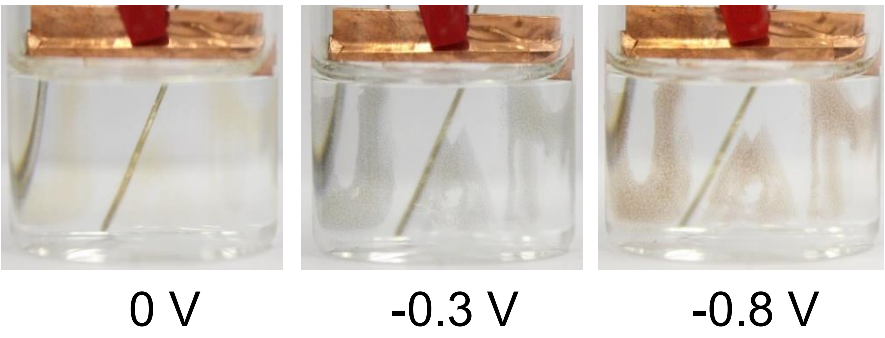

Figure S2. Electrochromic behaviour of patternable **poly-2** coated onto ITO glass in different redox states: dication form (left), radical cation (middle) after applying the potential of -0.3 V and natural form after of -0.8 V.

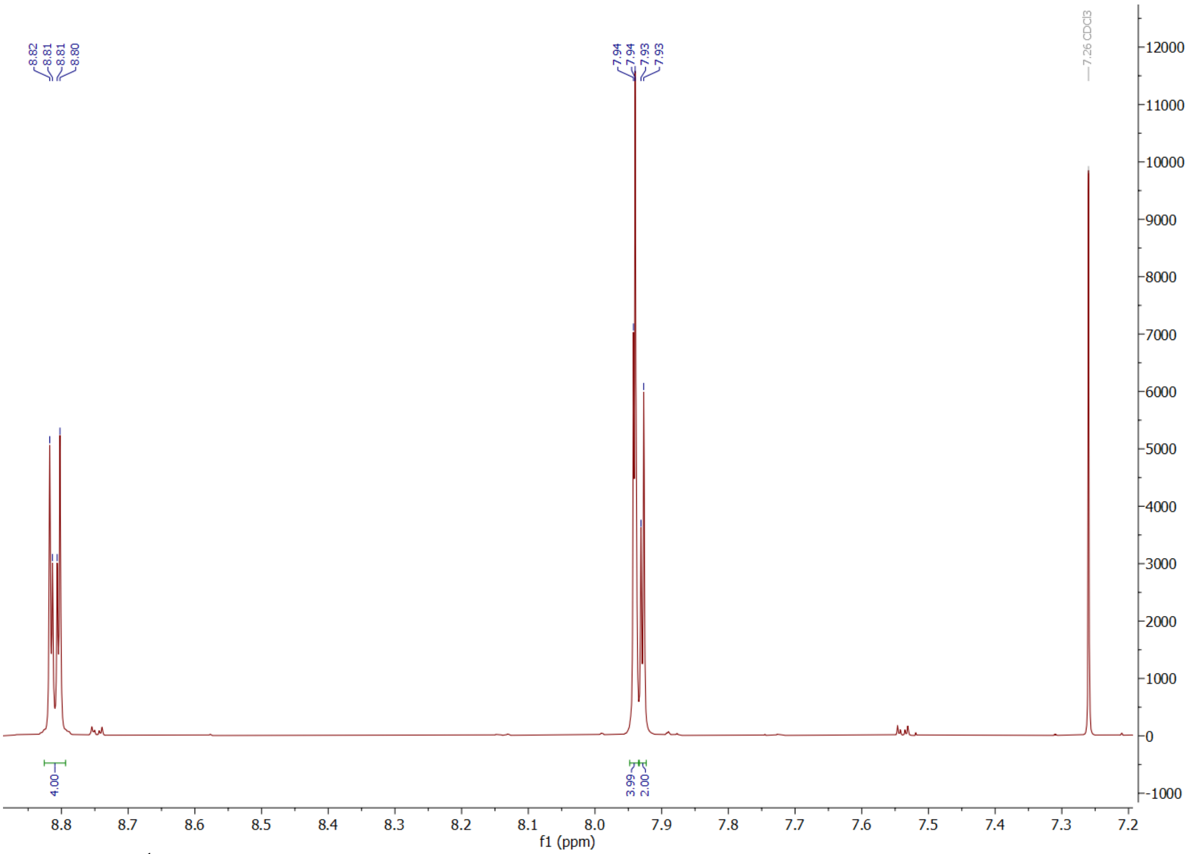

Figure S3. ^{1}H NMR spectra of compound **A** in $CDCl_3$

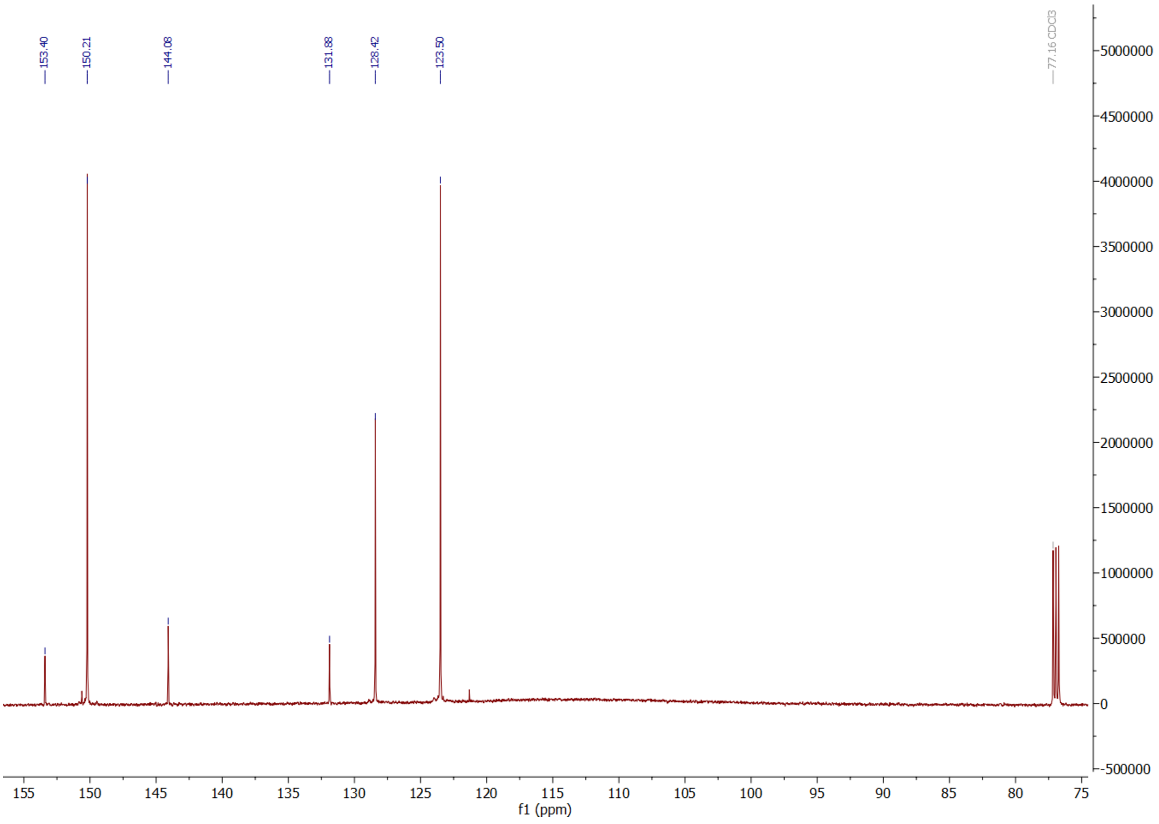

Figure S4. ^{13}C NMR spectra of compound **A** in $CDCl_3$

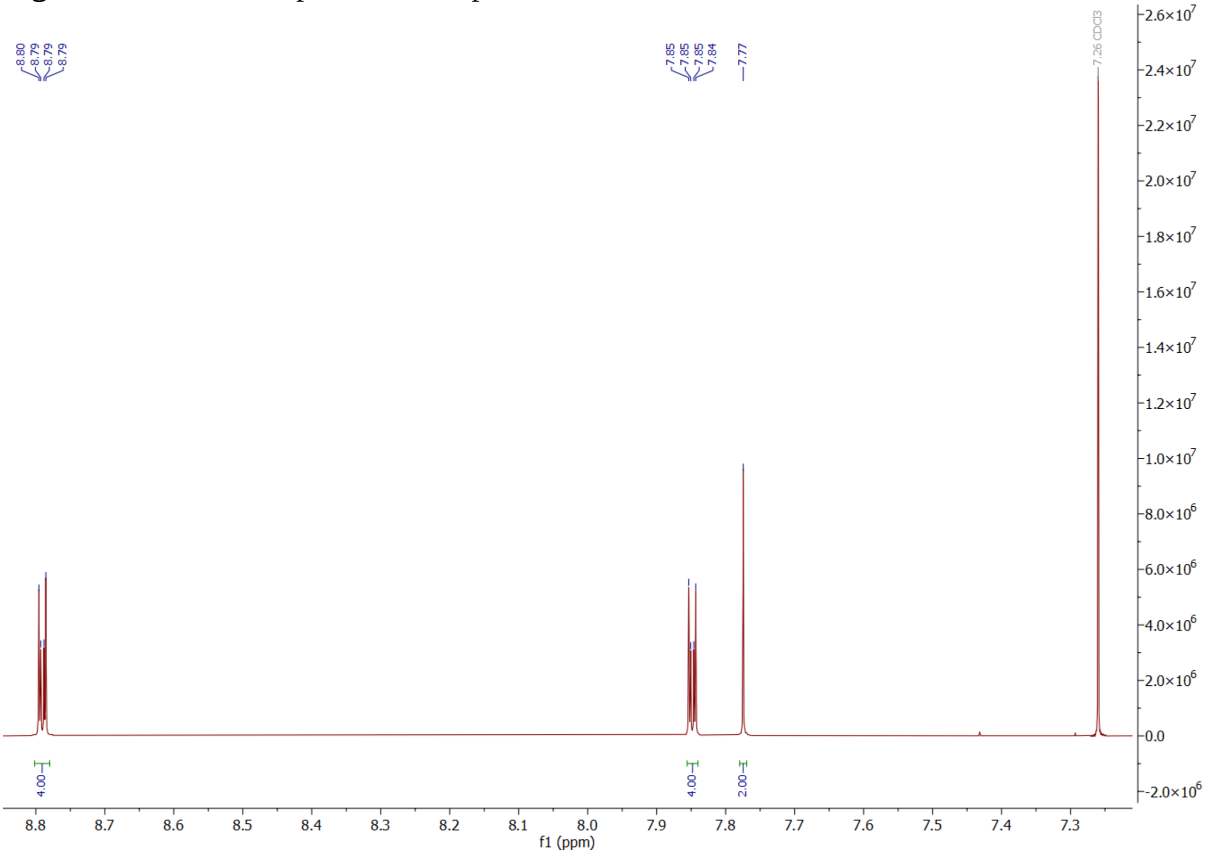

Figure S5. ^{1}H NMR spectra of compound **B** in $CDCl_3$

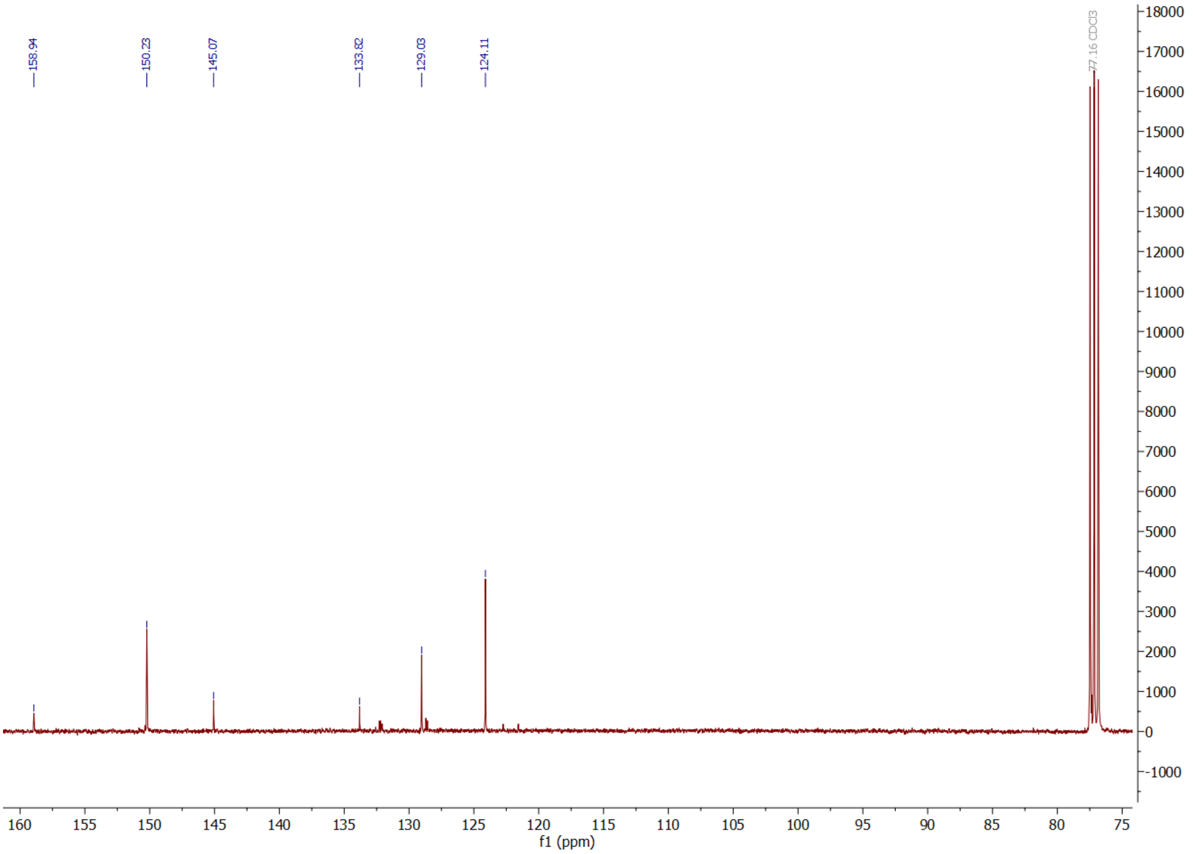

Figure S6. ^{13}C NMR spectra of compound **B** in $CDCl_3$

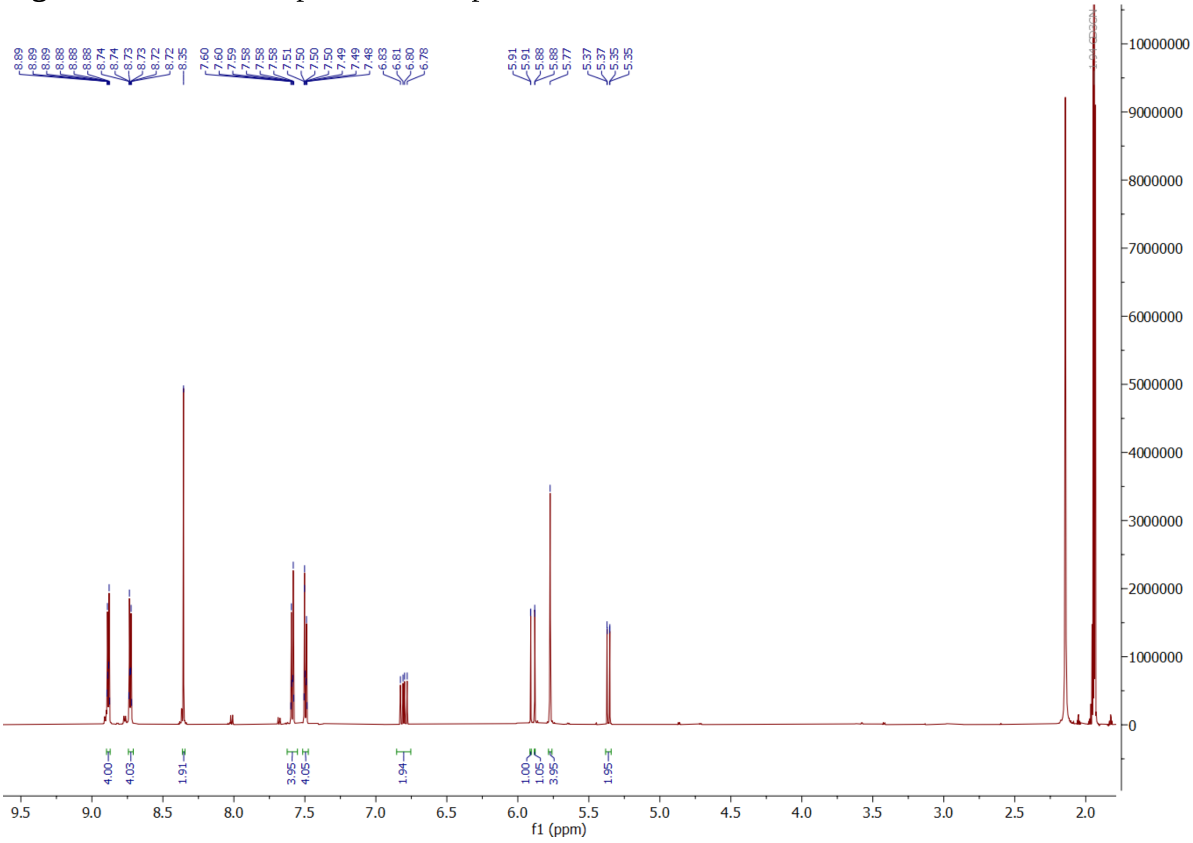

Figure S7. ^{1}H NMR spectra of compound **1** in CD_3CN

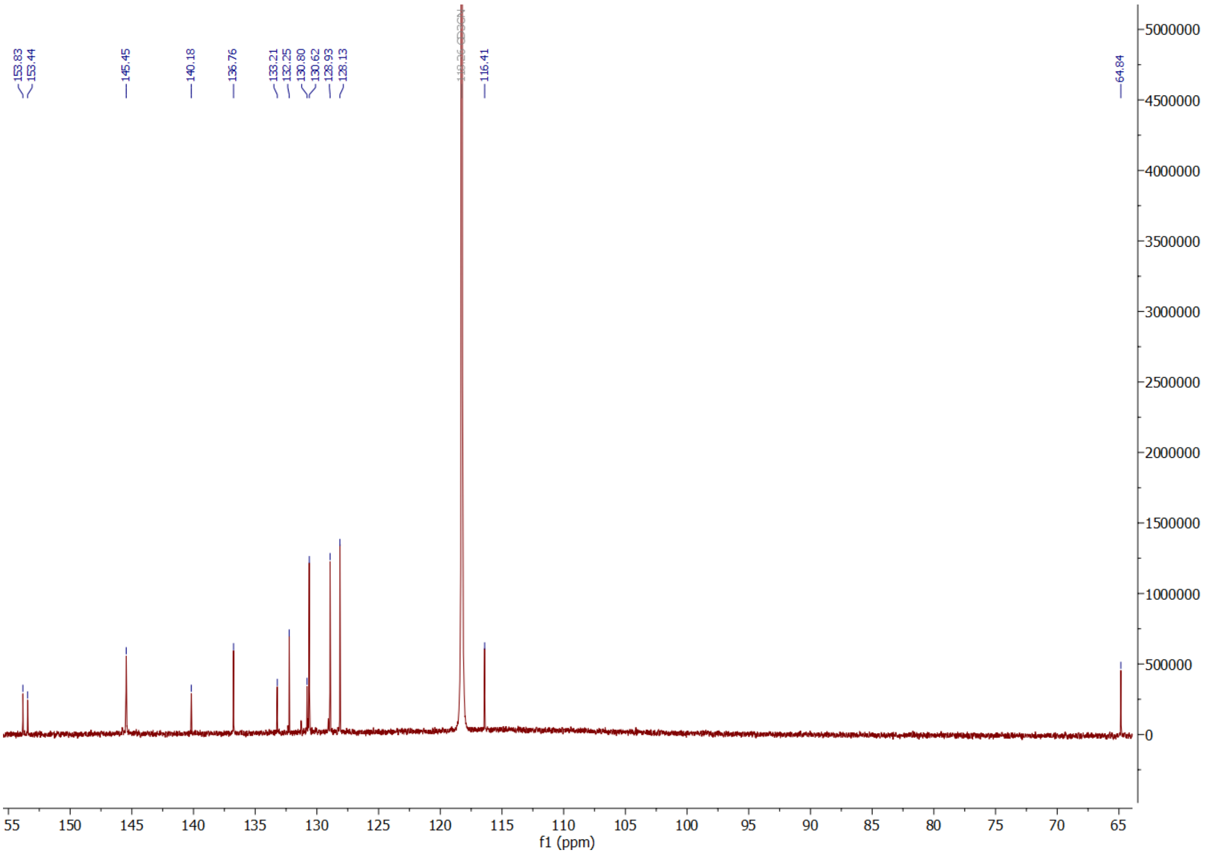

Figure S8. ^{13}C NMR spectra of compound **1** in CD_3CN

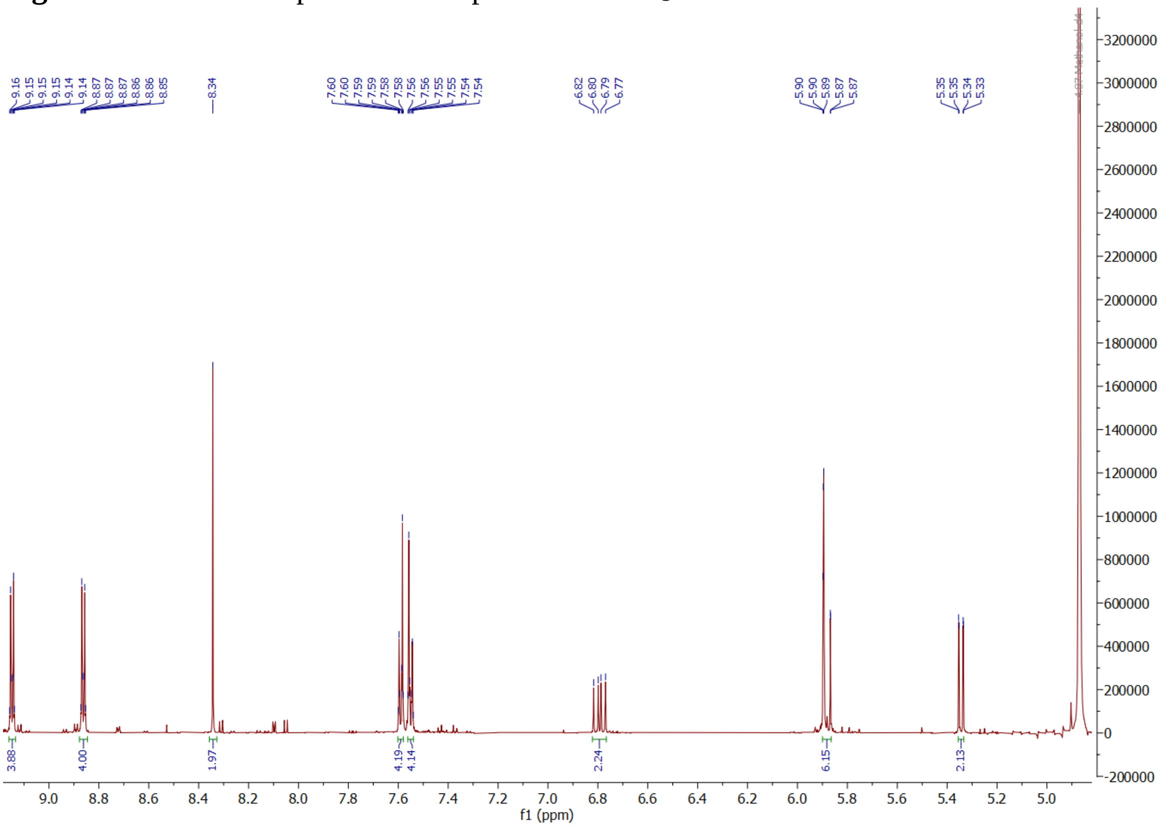

Figure S9. ^{1}H NMR spectra of compound **2** in CD_3OD

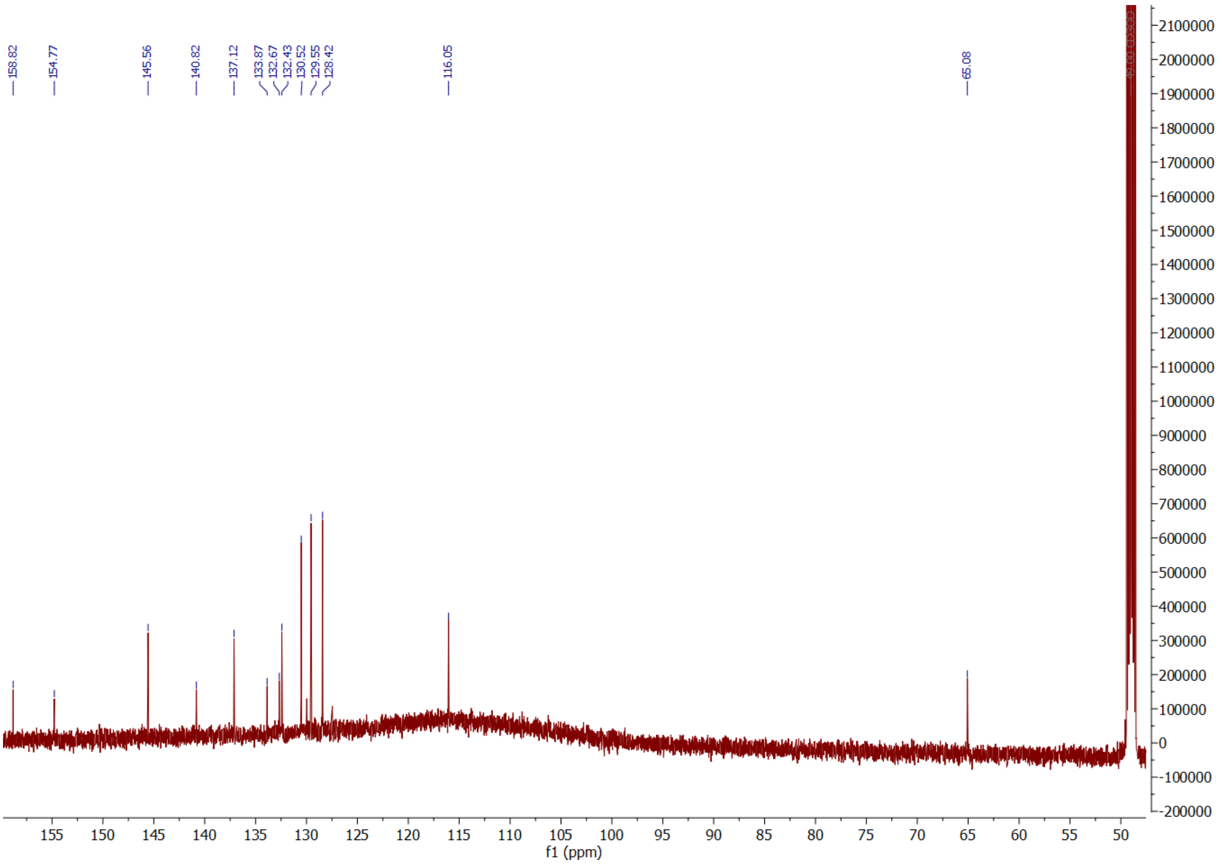

Figure S10. ^{13}C NMR spectra of compound **2** in CD_3OD

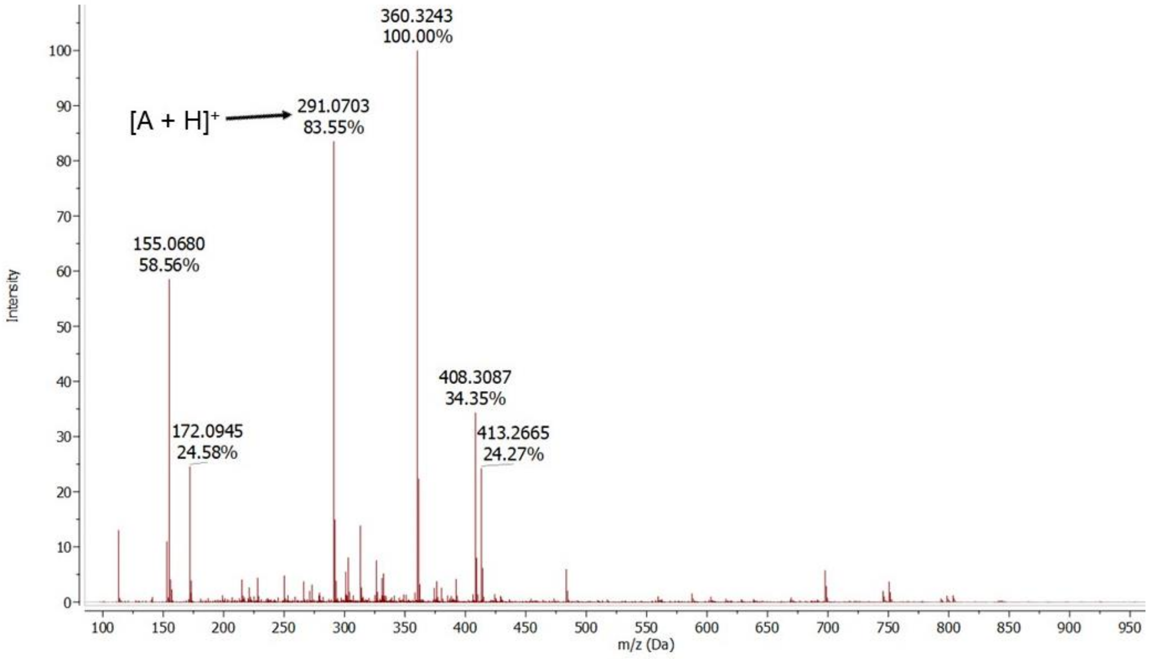

Figure S11. HR-ESI-MS spectra of compound **A**

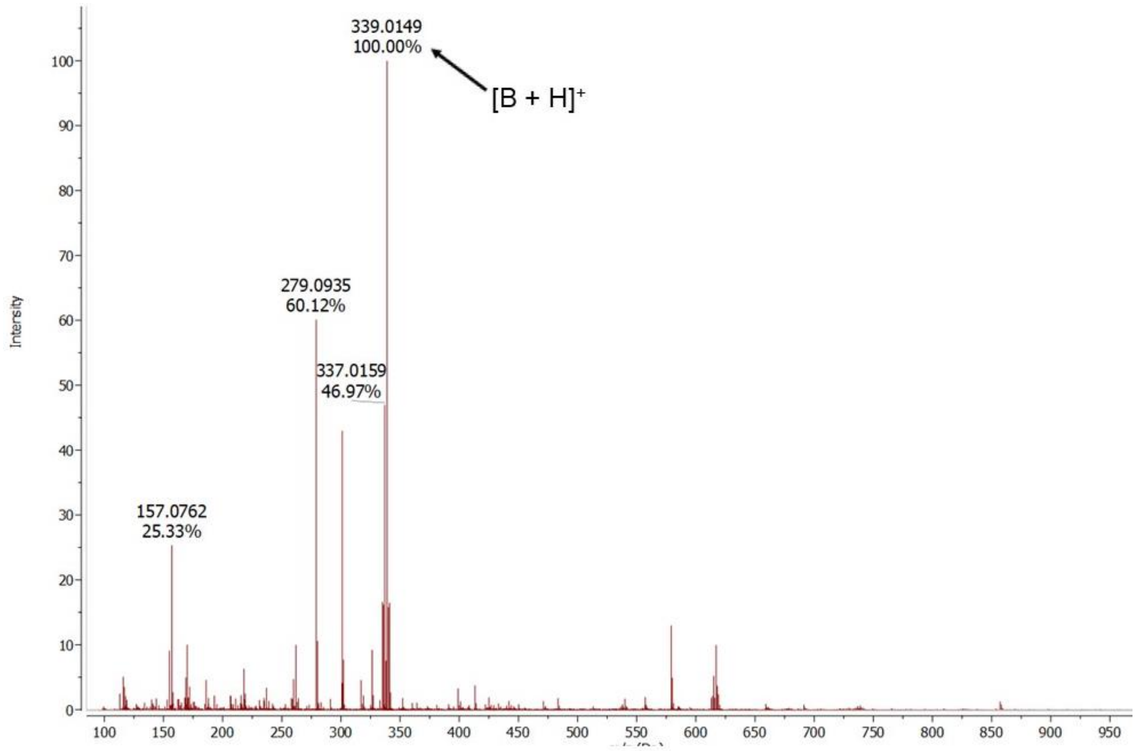

Figure S12. HR-ESI-MS spectra of compound **B**

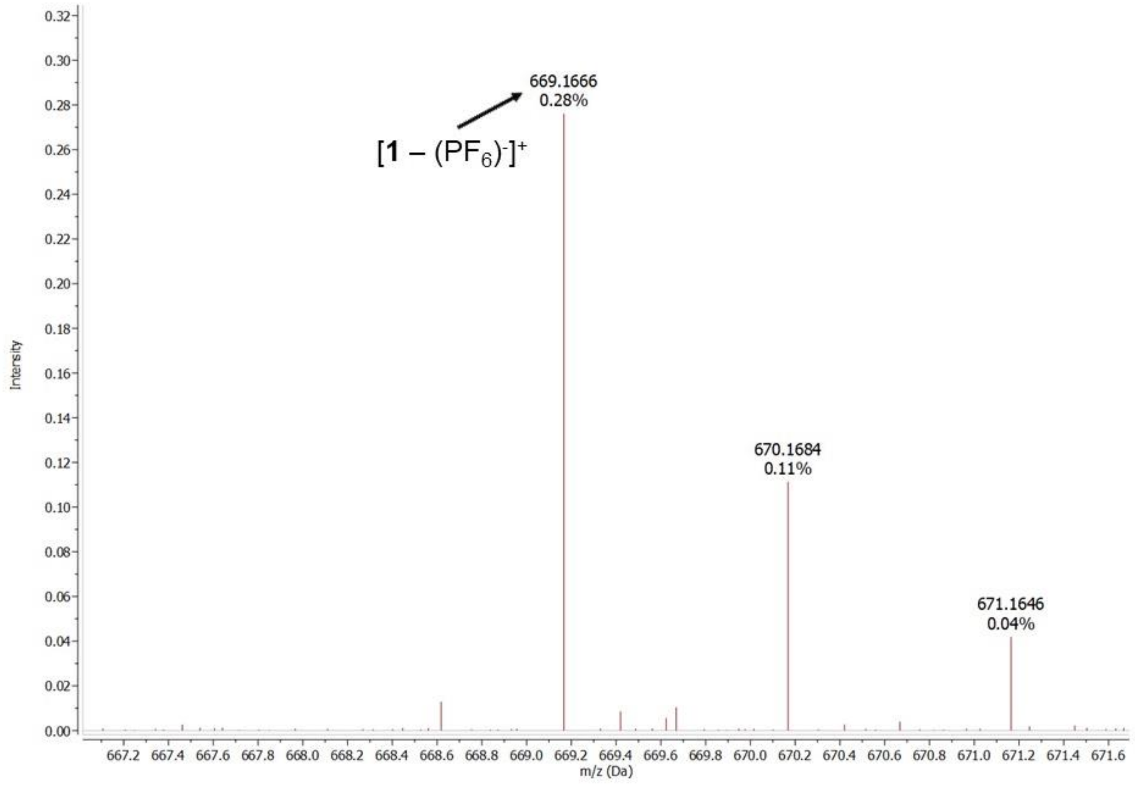

Figure S13. HR-ESI-MS spectra of compound **1**

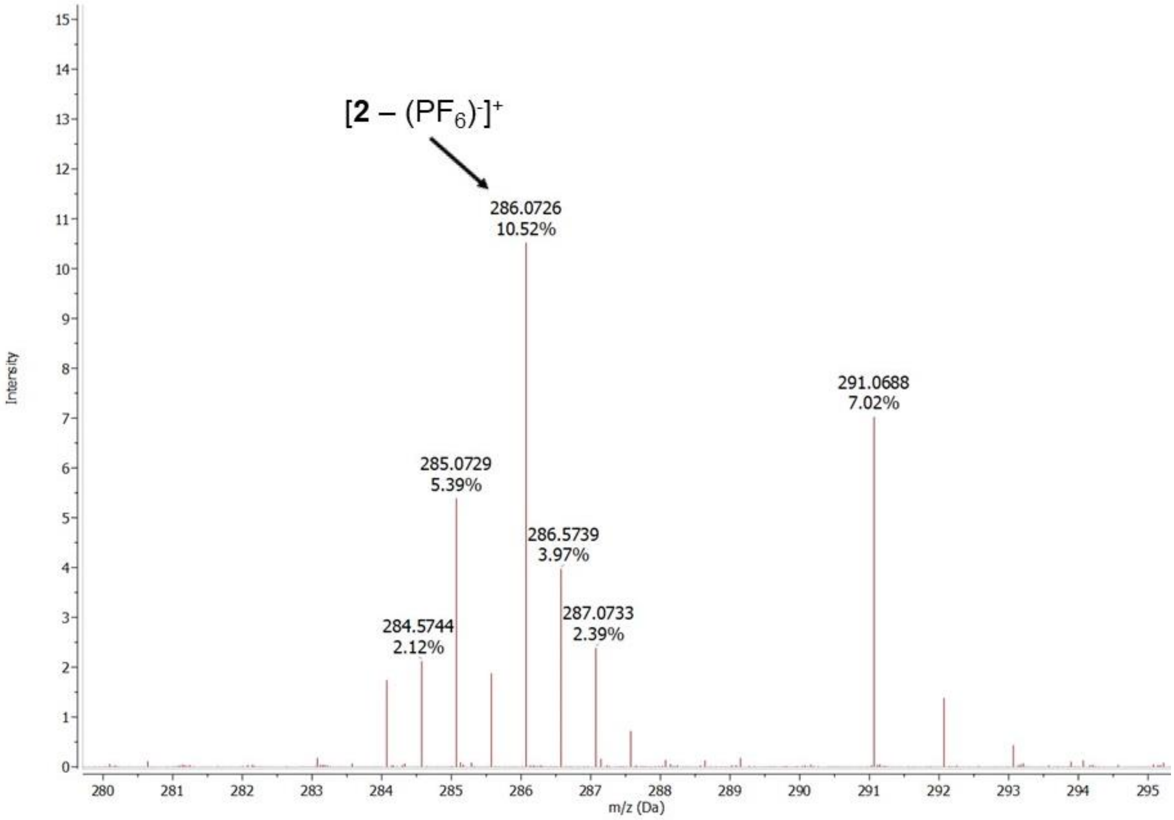

Figure S14. HR-ESI-MS spectra of compound **2**

References

1 M. Wałęsa-Chorab and W. Skene, *Electron. Mater.*, 2020, **1**, 40–53.

2 J. Kim, J. You, B. Kim, T. Park and E. Kim, *Adv. Mater.*, 2011, **23**, 4168–4173.

3 J. Kim, Y. Kim and E. Kim, *Macromol. Res.*, 2009, **17**, 791–796.

4 B. Ding, H. Gao, C. Wang and X. Ma, *Chem. Commun.*, 2021, **57**, 3154–3157.
